# From Sea to Skin: Is There a Future for Natural Photoprotectants?

**DOI:** 10.3390/md19070379

**Published:** 2021-06-30

**Authors:** Alfonsina Milito, Immacolata Castellano, Elisabetta Damiani

**Affiliations:** 1 Centre for Research in Agricultural Genomics, Department of Molecular Genetics, Cerdanyola, 08193 Barcelona, Spain; alfonsina.milito@cragenomica.es; 2 Department of Molecular Medicine and Medical Biotechnology, University of Naples Federico II, Via Pansini 5, 80131 Napoli, Italy; 3 Department of Biology and Evolution of Marine Organisms, Stazione Zoologica Anton Dohrn, Villa Comunale, 80121 Napoli, Italy; 4 Department of Life and Environmental Sciences, Polytechnic University of the Marche, Via Brecce Bianche, 60131 Ancona, Italy

**Keywords:** photoprotection, skin, marine natural products, solar radiation, antioxidants

## Abstract

In the last few decades, the thinning of the ozone layer due to increased atmospheric pollution has exacerbated the negative effects of excessive exposure to solar ultraviolet radiation (UVR), and skin cancer has become a major public health concern. In order to prevent skin damage, public health advice mainly focuses on the use of sunscreens, along with wearing protective clothing and avoiding sun exposure during peak hours. Sunscreens present on the market are topical formulations that contain a number of different synthetic, organic, and inorganic UVR filters with different absorbance profiles, which, when combined, provide broad UVR spectrum protection. However, increased evidence suggests that some of these compounds cause subtle damage to marine ecosystems. One alternative may be the use of natural products that are produced in a wide range of marine species and are mainly thought to act as a defense against UVR-mediated damage. However, their potential for human photoprotection is largely under-investigated. In this review, attention has been placed on the molecular strategies adopted by marine organisms to counteract UVR-induced negative effects and we provide a broad portrayal of the recent literature concerning marine-derived natural products having potential as natural sunscreens/photoprotectants for human skin. Their chemical structure, UVR absorption properties, and their pleiotropic role as bioactive molecules are discussed. Most studies strongly suggest that these natural products could be promising for use in biocompatible sunscreens and may represent an alternative eco-friendly approach to protect humans against UV-induced skin damage.

## 1. Introduction

Solar energy fuels life on Earth and, in part, in the oceans. Nevertheless, excessive sunlight can cause photodamage in photosynthetic organisms. In addition, 5% of the solar spectrum is constituted by ultraviolet radiation (UVR), which is partly shielded from gases in the atmosphere. Indeed, the ozone layer acts as a filter for UVR because it completely absorbs the UV-C component and 90% of UV-B rays. Therefore, only part of UV-B and UV-A reach the Earth and the sea and consequently can be deleterious for both terrestrial and aquatic organisms. 

The damaging effects of UVR can be mediated by the direct interaction of UV photons with DNA or indirectly by the production of reactive oxygen species (ROS), which can cause oxidative modifications to DNA and other macromolecules such as proteins and lipids [1]. 

Marine organisms living in the photic zone of the sea are constantly exposed to changes in light intensity and spectral composition; hence, they have evolved the ability to use solar radiation for survival but also to protect themselves from UVR-induced insults—for example, by triggering repair mechanisms after damage. 

Humans can take advantage and inspiration from strategies adopted by nature to counteract the negative effects of solar radiation, especially UVR. In particular, UVR can cause sunburn and erythema as a result of acute exposure, or even skin aging and cancer, along with harmful effects on the eyes and the immune system in the long term [2]. Indeed, although humans are endowed with endogenous mechanisms of defense against light-induced damage, such as melanin production, these mechanisms are insufficient to provide full protection from UVR. Moreover, in the last few decades, the thinning of the ozone layer due to increased atmospheric pollution has favored the penetration of UV-B rays in the atmosphere, thus increasing the exposure of humans to these rays.

Consequently, the World Health Organization (WHO) has laid out a set of recommendations regarding sun protection that include: (1) limiting sun exposure from 10 a.m. to 4 p.m., when UVR rays are stronger; (2) checking the UV index before planning outdoor activities; (3) staying under shady structures, such as trees or umbrellas, during the most intense hours, taking into consideration that they do not provide complete protection; (4) wearing protective clothes and accessories, such as hats and sunglasses; (5) using sunscreens; (6) avoiding sunlamps [3]. Thus, using sunscreens is one of the six key strategies to avoid UVR-induced damage. 

However, consumers are becoming increasingly aware of where the products they use come from, whether of synthetic or natural origin, and whether they are eco-friendly and eco-sustainable. Despite the knowledge that sunscreens are an undeniably important tool in the fight against skin cancer, their formulations may need to be improved to contain safer ingredients and to make them more water-resistant, particularly in view of the concerns raised on the potential eco-toxicity of some sunscreens to the marine environment as well as to human health. For instance, some sunscreens containing UV synthetic filters such as benzophenone-3 (oxybenzone) and octyl methoxycinnamate (octinoxate) have now been banned from sale and distribution in Hawaii as of 1st January 2021 for their suspected harm to coral reefs and marine life, and other USA locations have followed suit (Key West, U.S. Virgin Islands). Other nations, such as Palau, parts of Mexico, and the Caribbean islands of Bonaire and Aruba, have also put in place similar enforcements [4]. These UV filters, along with others, have also fallen under scrutiny for their potential toxicity concerning reproduction and development in various organisms [5]. Therefore, discovering and approving new sunscreen compounds with fewer downsides could alleviate concerns from all corners and increase sunscreen use. 

Turning to nature to replace synthetic chemicals is a growing trend in the pharmaceutical/cosmeceutical sector, where there is a surge in demand for valid alternatives of natural origin [6]. In this context, attention has recently been devoted to the photoprotective chemicals produced by the marine environment, either by extracting UV-filtering compounds from their natural sources or by engineering yeast or other organisms to produce them. Indeed, the huge and still underexploited marine biodiversity represents a yet untapped source of a wide range of naturally occurring UVR screening compounds, which can be used for cosmeceutical applications as eco-friendly and safer alternatives to synthetic UV filters.

The aim of this review is therefore to summarize the most recent literature on the molecular mechanisms evolved by marine organisms to counteract light-induced damage and to give a panorama on the diversity of photoprotective marine-derived compounds from which mankind could take inspiration to develop natural-based products that could be safer and more environmentally friendly.

## 2. The Photic Zone: Where Light Fuels Life in the Ocean

**Figure 1:**
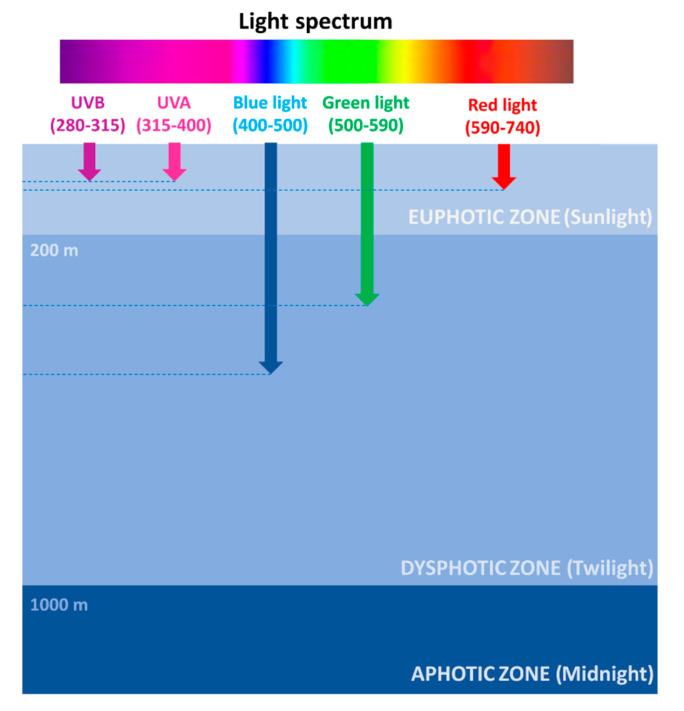
Light spectrum penetration at sea.

The photic zone is the uppermost layer of the sea, distinguishable in two main areas: the euphotic (or sunlight) zone, approximately 200 m deep, where sunlight allows phytoplankton to accomplish photosynthesis, and the dysphotic (or twilight) zone (200–1000 m deep), where sunlight rapidly decreases with depth and where photosynthesis cannot occur (Figure 1). 

However, the depth of the euphotic zone depends on the transparency of the water. If the water is very clear, light can penetrate deeper, while if the water is murky, the depth of the euphotic zone is reduced to only 50 feet (15 m). Ninety-five percent of photosynthesis in the ocean occurs in this zone, where the photosynthesis rate exceeds the respiration rate. Therefore, the high oxygen concentration fuels the life of 90% of marine organisms, including phytoplankton, such as dinoflagellates, diatoms, and cyanobacteria; zooplankton, such as copepods; and nekton, such as fish, squids, and crabs. 

In the ocean, exposure to sunlight is extremely variable along the water column, both in terms of photon flux density and spectral composition (Figure 1). Several factors are responsible for light intensity and spectrum variability at sea, including the geographical location, the day hours/season, the weather conditions, and the occurrence of organic matter. Red wavelengths (589–656 nm) are absorbed in the euphotic zone and are rapidly attenuated in the first water layers, thus reaching approximately 100 m in depth. Green wavelengths (479–579 nm) can penetrate until a depth of approximately 300 m and are used by algae for photosynthesis. Blue wavelengths (422–496 nm) can penetrate farther, being the only ones to reach the deep ocean, approximately 500 m in depth, and are mostly responsible for the color of the oceans [7,8]. The prevalence of blue/green light at sea has prompted marine organisms to sense and respond prevalently to these wavelengths, even though red/far-red sensing systems have also been described in diatoms [9].

## 3. Light Perception and Light-Driven Physiological Processes at Sea

Photosynthetic organisms mainly rely on light for their survival and they have evolved complex systems of photoperception and photoresponse. Blue light in particular is a key regulator of growth, development, and photosynthesis. It is sensed by blue light photoreceptors, consisting of flavoproteins belonging to the cryptochrome/photolyases family. They are known to mediate several light-induced responses, such as DNA repair, light perception, and the circadian clock [10,11]. However, several additional photosensors can be found in photosynthetic organisms, differing in the light absorption range, domain architecture, and bound chromophore. They are involved in the regulation of metabolism, cell cycle, and other important physiological processes [12]. 

However, light is not only an essential source of energy for photosynthetic organisms and, indirectly, for all life on Earth, but it can also be perceived and used by non-photosynthetic organisms. Marine animals, for example, use it for vision in the photic zone of the sea to escape predators or catch preys [13]. In most vertebrates, rod and cone photoreceptors mediate vision in low and high light, respectively [14]. These photoreceptors possess light-sensitive pigments, called opsins, whose number and type are extremely variable depending on the light habitat of the organism, allowing vision both in shallow waters, characterized by high light exposure, and in the dim light typical of deep oceans [15,16,17].

As mentioned above, the most dangerous portion of light belongs to the UVR spectrum (280–400 nm), which is able to reach the water column and induce responses in sea-inhabiting organisms. The degree of UVR penetration in water mainly depends on the optical properties of dissolved and suspended organic matter; hence, it is higher in clear open oceans than in turbid coastal waters [18,19]. Prolonged exposure to UVR can cause a variety of damage in living organisms, discussed in the next section. However, low levels have been reported to increase photosynthetic carbon fixation or even enhance primary production in certain conditions, and to influence the metabolism in photosynthetic organisms [20,21]. Furthermore, UV light is used for vision by some marine animals, such as coral fish, improving their ability to catch prey [22,23].

## 4. Photo-Damage/-Protection Mechanisms in Marine Organisms

**Figure 2:**
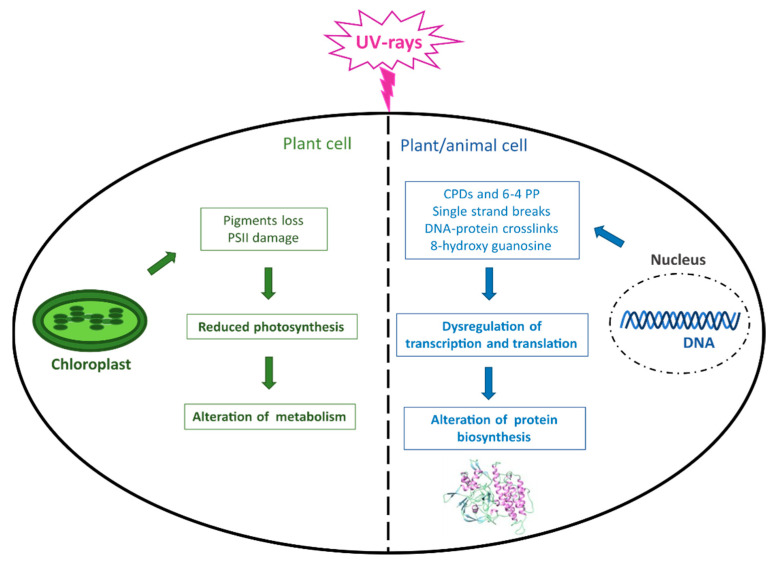
UVR-induced damage in living organisms. The figure summarizes the main effects caused by UVR occurring at the DNA level in both plant and animal cells and in chloroplasts in photosynthetic cells. CPDs = cyclobutane pyrimidine dimers; 6–4 PP = pyrimidine (6–4) pyrimidone photoproducts; PSII = Photosystem II.

Although light is essential for life on Earth and in the oceans, photosynthetic organisms can be harmed by an excess of photons reaching the photosynthetic apparatus, whose damage can result in a decrease in photosynthesis and metabolism (Figure 2). Moreover, the major macromolecules, including proteins, lipids, and especially DNA, can be damaged by both direct UVR and consequent ROS production, which ultimately leads to altered protein biosynthesis and crucial cellular functions not only in plant but also in animal organisms (Figure 2). 

Such an environmental constraint has forced them to evolve a complex system of photoprotective mechanisms and antioxidant enzymes and molecules, in order to counteract light-dependent stress. For instance, the first defense mechanism used by algae against high light is the dissipation of excess light into heat through non-photochemical quenching (NPQ) of chlorophyll fluorescence. This mechanism allows algae to prevent photodamage [24]. In particular, the increased proton flux leads to acidification of the thylakoid lumen inside chloroplasts, with consequent activation of the xanthophyll cycle, a key player in the NPQ process [25]. In addition to these short-term responses, cells have adopted a variety of long-term responses to counteract light stress, including several integrated signaling pathways and associated changes in gene expression. In particular, variations in the state of chloroplasts are sensed by the nucleus through a “retrograde signaling” in which the tetrapyrroles of chlorophyll seem to play a central role [26]. 

However, despite such protective mechanisms, high light exposure can ultimately lead to the formation of ROS. Algae possess a complex enzymatic and non-enzymatic defense system to prevent photo-oxidative damage to cellular components. Enzymatic antioxidant protection includes three types of superoxide dismutases possessing different metal cofactors (CuZn-SOD, Fe-SOD, Mn-SOD), catalase, ascorbate peroxidase, glutathione peroxidase, while the non-enzymatic defense system includes ascorbic acid, glutathione, tocopherols, carotenoids, polyphenols, phycobilin proteins, dimethylsulfide/dimethylsulfoxide, sulfated polysaccharides [27], and sulfur-containing histidines such as ovothiols [28,29]. 

As mentioned earlier, the UV portion of light is able to reach water bodies and can represent a source of damage for their inhabitants. UVB (280–315 nm) in particular can induce damage to DNA, through the generation of cyclobutane pyrimidine dimers (CPDs) and pyrimidine (6–4) pyrimidone photoproducts (6–4 PP), while UVA (315–400) mainly induces indirect oxidative damage to DNA in terms of single-strand breaks, DNA–protein crosslinks, and 8-hydroxy-guanosine (8-oxoG) [30]. These photoproducts can inhibit DNA replication and cell cycle progression, threatening, for example, phytoplankton growth and photosynthesis, although the sensitivity to UVR can vary among different species [31,32,33,34]. UVR are not only dangerous for photosynthetic organisms but they can cause damage to a wide range of living organisms, from bacteria to higher vertebrates, and their effects can change depending on other environmental conditions [35]. For instance, UVR negatively affects the viability and development of invertebrates and fish, whose early developmental stages are generally more sensitive [36,37]. Indeed, CPDs formation in sea urchin embryos and larvae inhibits their development, decreasing population survival and fitness [38].

In addition to the above-mentioned enzymatic and non-enzymatic antioxidant systems used by photosynthetic organisms to counteract light stress, marine animals have adopted some UVR-avoiding strategies, such as living in deeper water. Several organisms have also adapted by synthesizing natural UV filters, such as melanin and mycosporine-like amino acids (MAAs) [39,40], or by acquiring screening compounds, such as scytonemin, flavonoids, and carotenoids from other organisms via their diet or by symbiosis.

## 5. UVR Detrimental Effects on Humans

All living organisms can be exposed to the harmful effects of UVR and humans are not an exception. Indeed, even though UVB rays are useful for vitamin D biosynthesis in human skin [41], they can lead to a variety of dysfunctions, especially in certain human organs that are mostly exposed to UVR, such as the eyes and skin. While the only clinically relevant effect of excessive UVR exposure on the eyes is represented by a temporary inflammation of the corneal epithelium, generally appearing 6–12 h after exposure and resolving in around 48 h without long-term effects, the UVR-induced photodamage in the skin is quite complex [42]. 

**Figure 3:**
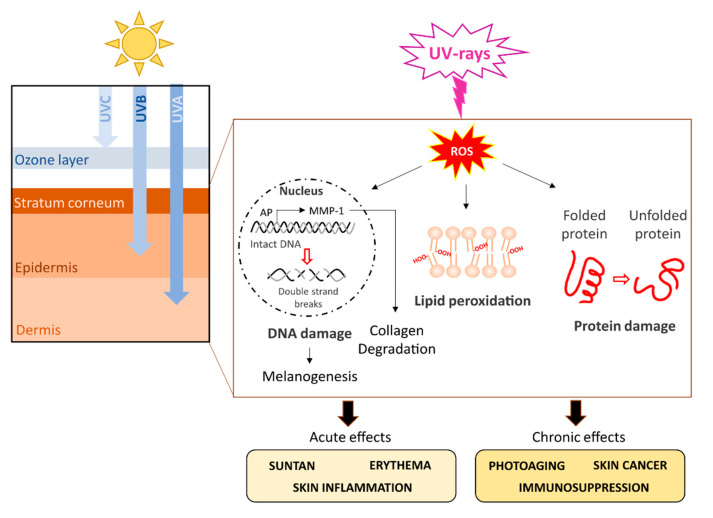
Major UVR-induced damage in humans. The figure summarizes the main direct and ROS-mediated effects caused by UVR in human skin. Abbreviations: AP = activator protein; MMP-1 = matrix metalloproteinase-1.

The degree of skin penetration of UVR depends on the wavelength (WL). In particular, UVB rays, having a shorter WL (280–320 nm), are absorbed by keratinocytes in the stratum corneum of the epidermis, whereas UVA rays, having a longer WL (320–400 nm), are able to penetrate deeper into the dermis [1] (Figure 3). 

The first acute reaction to UVR is a suntan due to DNA photodamage and the consequent stimulation of repair mechanisms and melanogenesis [43,44]. High levels of UVR (especially UVB) can cause skin inflammation, erythema, edemas, blisters, and sunburn cells, which undergo apoptosis [45]. Moreover, UVR can lead to a suppression of local and systemic immunity, thus exposing the body to several types of infections [46]. Chronic UVR effects include skin cancer, photoaging, and immunosuppression [47,48,49] (Figure 3). 

Although humans produce protective molecules such as melanin, which is, to some extent, able to protect DNA from photodamage, this is often not enough for providing sufficient protection against prolonged or excessive exposure to UVR. To help the skin to protect the body against excessive UVR, the cosmetic and pharmaceutical industries have developed several products mainly for topical application, such as sunscreen formulations.

## 6. UV Filter Properties

Typically, a sunscreen is conceived as a formulation containing UV chemical filters, which can be either inorganic, such as zinc oxide (ZnO) and titanium dioxide (TiO_2_), or organic, such as cinnamates and benzophenones [50,51]. Conceptually, the absorption of the UVR photons’ energy causes a photochemical excitation of the filter molecule to a higher energy excited state. As the excited molecule returns to its ground state, the excess energy is partially dissipated as heat. In some cases, the absorbed energy may be transferred to a receptor molecule, or emitted in the visible region and perceived as fluorescence, or it may be emitted in the longwave UVR (380–450 nm), which may cause a photochemical reaction such as cis-trans or keto-enol isomerizations in a fraction of the filter molecules. These photochemical reactions, in some cases, may give rise to instability in the filter molecule, with chemical bond cleavage and formation of photodegradation products. This is a highly undesirable property for a UV filter since the loss in absorption efficacy defeats the purpose of a UV filter for sunscreen use. An efficient UV filter should be able to return to its ground state in its original form such that it may be available to absorb another photon. This guarantees photoprotection throughout the entire duration of UV exposure [52].

Organic UV filters used in sunscreens are powerful photochemicals whose behavior is closely related to their molecular structure. They typically contain a chromophore, which is usually an aromatic molecule conjugated to carbonyl groups. In general, an increased number of conjugated double bonds and resonance structures stabilizes the excited state, shifting the absorption spectrum of the compound to longer wavelengths. Instead, inorganic filters such as ZnO and TiO_2_, commonly used in sunscreen formulations, besides absorbing UV light, also possess scattering and reflecting properties. They are generally considered more natural and benign than organic UV filters, despite the fact that they contain a wide array of coatings, additives, predispersants, and dispersion enhancers. Furthermore, because of their particulate nature, which causes the so-called “whitening” phenomenon upon application, they are nowadays generally micronized to improve their cosmetic appearance [53]. 

There is a growing body of evidence suggesting that the synthetic UVR filters such as octocrylene, benzophenone-4, ethyl 4-aminobenzoate, 3-benzylidene camphor, and TiO_2_ nanoparticles may cause damage to the marine environment, due to their widespread use on sea resorts. Several negative effects include the bioaccumulation of filters in many different species, hormonal changes and endocrine disruption in fish, abnormal development in sea urchins, and the bleaching of corals [54,55,56,57]. 

In this context, sunscreen compounds of natural origin may provide an eco-friendly alternative to synthetic products. In particular, the marine ecosystem offers an extremely rich biodiversity of life forms, most of which are still underexploited as natural sources for cosmeceutical products [58]. Several classes of marine-derived molecules will be here discussed, including their chemistry, occurrence, natural/physiological functions, and bioactivity in in vitro mammalian cells, with a special focus on their photoprotective abilities.

## 7. Mycosporine-Like Amino Acids (MAAs)

**Figure 4:**
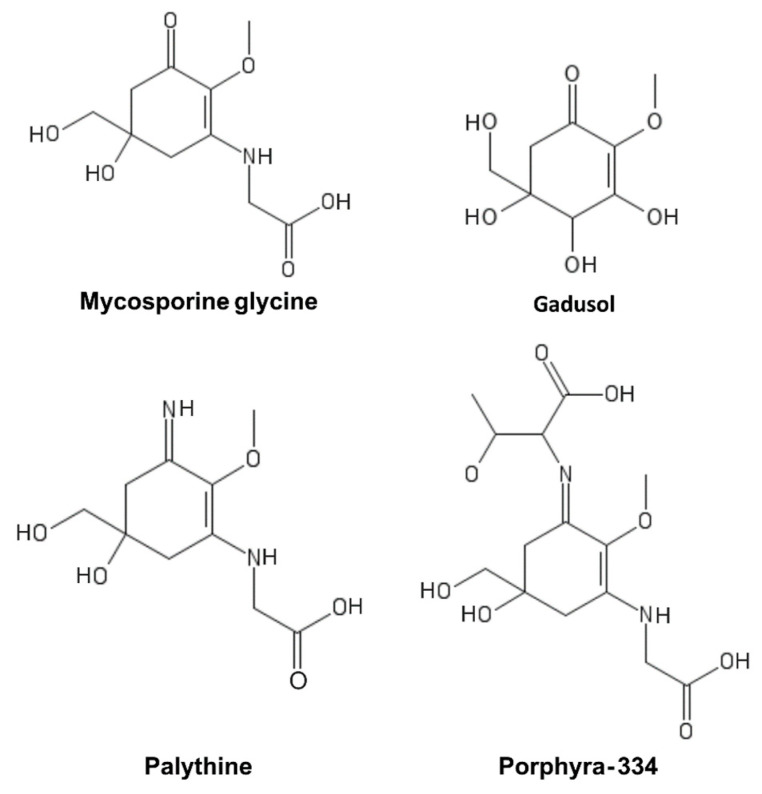
Mycosporine-like amino acids (MAAs).

MAAs are a heterogeneous group of hydrophilic molecules (<400 Da). Their structure consists of a cyclohexanone or cyclohexenimine ring system conjugated to an amino acid or its imino alcohol [59]. Representative MAAs are reported in Figure 4. 

They absorb in the 310–362 nm range with a high molar extinction coefficient and are produced by organisms that are particularly exposed to UV light, such as cyanobacteria [60], micro- and macroalgae [61,62], dinoflagellates [63], and fungi [64]. Their biosynthesis is mainly ascribed to the shikimate pathway, although the pentose-phosphate pathway has also been suggested to be involved in their biosynthesis [65,66]. Aromatic amino acids and MAAs in marine organisms have been generally hypothesized to be of dietary origin since most metazoans lack genes coding for the enzymes of the shikimic acid pathway [64,67,68]. However, previous studies have suggested that cnidarians, including nonsymbiotic corals and anemones, are capable of synthesizing these essential amino acids [68,69,70,71]. Starcevic and colleagues [70] provided evidence for the horizontal transfer of both bacterial and dinoflagellate ancestral genes of the shikimic acid pathway into the genome of *Nematostella*. Moreover, besides corals, the endogenous synthesis of gadusol, the main MAAs precursor, has been discovered to occur in fish through an alternative pathway, which is also present in amphibians, reptiles, and birds [72,73]. 

MAAs play an important sunscreen role in producer organisms due to their properties, which include strong UVA absorbance, photostability, and widespread distribution in cell cytoplasm [68]. In addition, MAAs possess high stability under different pH and temperature values, representing a key advantage for their use as UV filters [74]. Furthermore, MAAs also function as strong antioxidants and additional roles have been suggested in cellular osmotic regulation during salt stress or as nitrogen reservoirs during nitrogen limitation [63,75]. Several studies have reported the ability of MAAs to protect against DNA damage induced directly by UVR and indirectly by ROS production triggered either chemically or by UVA radiation [76,77,78]. MAAs have also shown promising antioxidant, anti-inflammatory, and anti-aging activities in in vitro studies on human cells. In particular, their anti-aging properties were shown to be partially mediated by a direct inhibitory activity on protein glycation and collagenase enzymes [79]. To date, only a few products that include MAAs in their formulations, such as Helioguard^®^365 and Helionori^®^, have been released [80,81]. Among them, palythine, extracted from the red alga *Chondrus yendoi* has recently been incorporated as an active ingredient in the skin care product line Aethic^®^, and launched on the market in 2021 as Dermagie. These algae can be harvested year-round and can protect human skin cells from UVA and UVB damage under artificial sunlight as intense as at noon during summer in Arizona [78]. Nevertheless, further studies are necessary to fully assess the efficacy of MAAs for application in topical formulations [81,82].

## 8. Scytonemin

**Figure 5:**
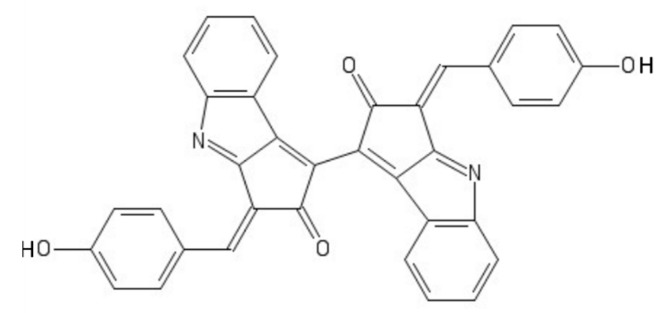
Scytonemin.

Scytonemin is a hydrophobic dimeric molecule composed of an indolic and phenolic subunit, linked by a carbon–carbon bond [83]. Its complex ring structure (Figure 5) allows a wide absorption range not only in the UVB but also in the UVA range, reaching a maximum of 370 nm [83]. 

It is produced by some species of cyanobacteria, such as *Scytonema sp*., having the ability to produce extracellular polysaccharides (EPS) and living in harsh environments typically characterized by desiccation periods, high temperatures, and nutrient limitation [84,85,86,87]. Scytonemin-producing cyanobacteria secrete this molecule in the extracellular matrix, where it acts as a physical barrier against UVA and as an antioxidant molecule against ROS produced following UVA exposure or several other abiotic stressors [86,88,89]. Scytonemin biosynthesis includes the involvement of enzymes from the shikimate pathway, tyrosine and tryptophan biosynthesis, and two additional specific enzymes called ScyB and ScyA [90]. 

The high extinction coefficient (ε = 250 Lg^−1^cm^−1^ at 384 nm) and the exceptionally high photostability make scytonemin an efficient photoprotective chemical [91]. The UV-protective abilities of scytonemin extracted from cyanobacterial biofilm have been demonstrated in UV-irradiated *E. coli* cells [92].

In addition, scytonemin is also endowed with other biological activities, acting as an antioxidant [86], anti-inflammatory [93], and antiproliferative agent [94,95,96], and as a calcium-channel blocker [97]. Moreover, due to its presence in organisms able to survive in extreme environments, it has been proposed as a biomarker for the identification of life on extraterrestrial planets [98,99]. 

## 9. Polyphenols

Polyphenols are a large class of secondary metabolites characterized by the presence of several phenol rings. Among them, phenolic acids and flavonoids are the most abundant [100]. They are mainly found in plants, including fruits, vegetables, tea, and other plant-derived beverages. Due to their pleiotropic properties, they are endowed with numerous beneficial properties for human health, ranging from the prevention of cardiovascular diseases, metabolic syndrome, and neurodegenerative disorders, to anti-aging, anticancer, and antimicrobial activities [101,102,103]. They have also recently been proposed as an alternative co-adjuvant therapy against coronavirus infections, or in combination with classical antiviral drugs [104].

Besides terrestrial sources, marine organisms are also able to produce polyphenols, especially flavonoids. Most marine flavonoids have been found in seagrasses and halophytes but they have been identified also in mangroves, bacteria, algae, fungi, and corals. Some of these molecules present unique substituents compared to their terrestrial counterparts, such as the occurrence of sulphate, methyl, chlorine, amino acids, and aminodeoxy sugar groups [105]. Marine polyphenols have been shown to have the classical pharmacological activities ascribed to those of terrestrial origin, such as antioxidant, antidiabetic, anticoagulant, antimicrobial, and anticancer properties, and, in addition, they possess antifouling and antifeedant activities [105].

**Figure 6:**
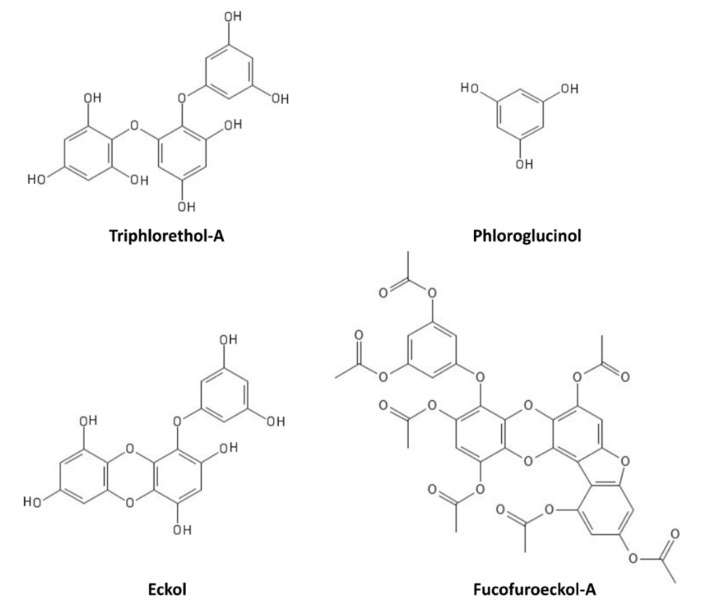
Marine polyphenols with UVR-protective properties.

Photoprotective activities have also been described for polyphenols, whose action is either direct, due to their UV absorption ability, or indirect, thanks to their antioxidant/scavenging function and consequent regulation of different signaling pathways [106,107]. For instance, polyphenols extracted from brown algae have been reported to exert protection against UVB-induced damage in human cells and in an in vivo zebrafish model [108,109,110,111]. In particular, triphlorethol-A (Figure 6) protects human keratinocytes (HaCat) against UVB-induced damage, by exerting a UV-filtering and antioxidant function, as well as by inhibiting the caspase pathway [108]. Phloroglucinol (Figure 6) also showed UVB protection properties in HaCat cells, through the inhibition of key molecular mediators of UVB-induced photoaging, including MMP-1 (matrix metalloproteinase-1) activity, Ca^2+^ levels increase, MAPK (mitogen-activated protein kinase) phosphorylation, c-Fos and phospho c-Jun expression, and AP-1 (activator protein-1) binding to the MMP-1 promoter [109]. Other polyphenols, such as the phlorotannin eckol (Figure 6), protect human keratinocytes against UVB-induced oxidative stress by scavenging ROS and reducing apoptosis by inhibiting mitochondrial membrane disruption, thus ameliorating injury to cellular components [110]. Dieckol (composed of two molecules of eckol) showed a particularly strong UVB-protective ability both in HaCat cells and in live zebrafish, where it reduced ROS, NO (nitric oxide), and cell death [111]. Another derivative of eckol, fucofuroeckol-A (Figure 6), showed photoprotective properties since it reduced the UVB-induced allergic reaction in RBL-2H3 mast cells, leading to the suppression of mast cell degranulation and decreasing histamine release and Ca^2+^ levels [112]. Because of these photoprotective properties, polyphenols could hence be repurposed for cosmetics development in the sunscreen sector [113].

## 10. Carotenoids

**Figure 7:**
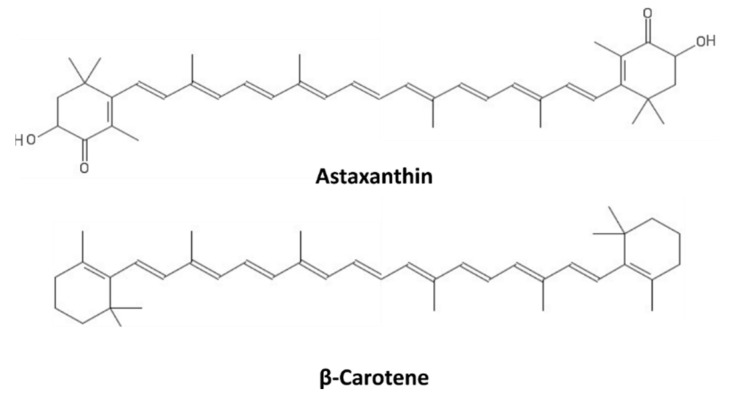
Carotenoids.

Carotenoids are a broad class of tetraterpenoids formed by 5-carbon isoprene units, polymerized to generate 40-carbon structures containing up to 15 conjugated double bonds. They include two major categories, carotenes and xanthophylls, with the latter possessing oxygen atoms in their structures. Some carotenoids consist of 6-carbon rings at one or both ends of the molecule; in the case of the xanthophylls, they possess oxo, hydroxy, or epoxy substitutions [114]. The structures of β-carotene and astaxanthin, as representatives of carotenes and xanthophylls, respectively, are shown in Figure 7. 

Carotenoids absorb in the blue-green region of the light spectrum (450–550 nm), and in photosynthetic organisms, they act as both light-harvesting molecules and as photoprotective agents through their ROS scavenging activity, thus contributing to most of the NPQ [115]. While photosynthetic organisms, including unicellular cyanobacteria and microalgae or multicellular algae and plants, are natural producers of carotenoids, marine animals can only acquire them through the diet or symbiotic relationships, such that they can also benefit from the valuable properties of carotenoids, including photoprotective action [116]. Carotenoids are mainly known as precursors of vitamin A biosynthesis in humans; however, they are endowed with multiple beneficial activities against neurodegenerative diseases, cancer, and cardiovascular pathologies [117]. Several studies have reported promising photoprotective activities of carotenoids against UV light-induced erythema [118]. In this process, the singlet oxygen (^1^O_2_) quenching activity is crucial, but they also activate different signaling pathways. β-Carotene, for instance, induces gene expression changes in human keratinocytes, resulting in decreased extracellular matrix degradation and increased cell differentiation. Both these effects were enhanced following keratinocyte irradiation with UVA light [119]. These mechanisms of action highlight the potentialities of β-carotene as a protective agent against photoaging and skin diseases, such as skin cancer and psoriasis. Two marine carotenoids, astaxanthin and fucoxanthin, also revealed promising anti-inflammatory and immunostimulant activity in addition to their protective properties against DNA damage and oxidative stress [120]. Astaxanthin, especially, has been tested in in vivo studies and clinical trials. Indeed, the application of a liposomal preparation of astaxanthin on the dorsal skin of Hos:HR-1 hairless mice prevented wrinkle formation induced by UV exposure [121] and its effects were confirmed by additional studies on animal models [122,123]. Moreover, astaxanthin supplementation in humans significantly increased their minimal erythema dose [124], defined as “the smallest UV dose that produces perceptible redness of the skin (erythema) with clearly defined borders at 16 to 24 h after UV exposure”, and different other clinical trials highlighted the prevention and curing abilities of astaxanthin against photoaging [125,126,127,128].

## 11. Sulfated Polysaccharides

**Figure 8:**
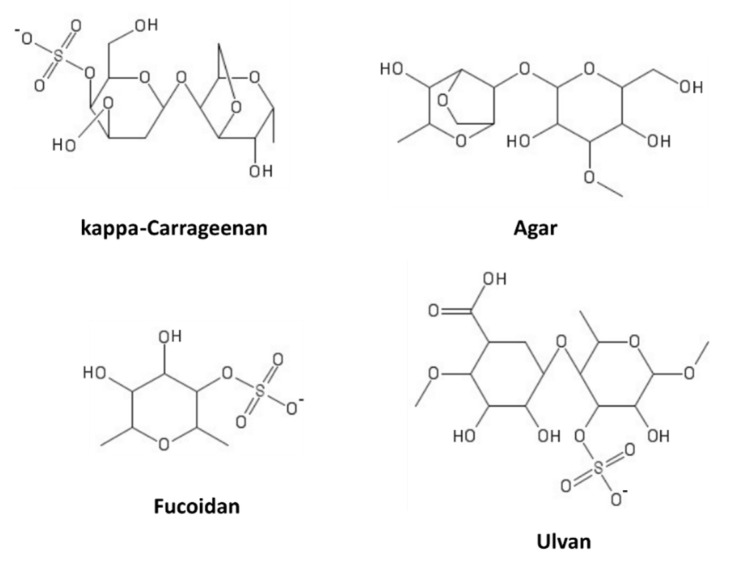
Marine sulfated polysaccharides.

Sulfated polysaccharides (SPs) consist of more than 10 monosaccharides, possessing sulfate groups on the hydroxyls of sugar units. They are present in several natural sources, such as plants, or can be obtained by chemical substitution of non-sulfated polysaccharides [129]. Marine macroalgae (seaweeds) are rich sources of these compounds. Based on their sugar composition, stereochemistry, and glycosidic linkages, algal SPs can be distinguished in sulfated galactans (carrageenans and agarans, produced by red algae), ulvans (in green algae), and fucans (in brown algae) [130]. Some representatives of these classes are shown in Figure 8. 

Some of them play structural roles in the cell wall composition, while others represent a sort of energy storage. Little is known about other physiological functions of SPs [131,132]; for example, they have been suggested to confer resistance against desiccation and osmotic stress in marine plants [133]. In addition, SPs have also been found in marine sponges, where they are presumably involved in species-specific cell aggregation and structural integrity, resembling the structural function of glycosaminoglycans in the connective tissues of mammals [134]. For their gelling properties, SPs are used in drug delivery systems, while, for their numerous bioactivities, including their ability to modulate metabolism and the immune system, and their anticancer, antimicrobial, and anticoagulant properties, they possess many applications in the cosmetic, nutraceutical, and pharmaceutical industries [135,136]. 

SPs do not absorb in the UV region; nevertheless, they showed strong UV-protective activities in in vitro and in vivo studies [137,138,139]. The protective effects of SPs against UVB-induced skin damage were tested in vitro in human dermal fibroblasts. SPs significantly reduced intracellular ROS levels and improved the viability of UVB-irradiated cells in a dose-dependent manner. Furthermore, they significantly inhibited intracellular collagenase and elastase activities, prevented collagen synthesis, and reduced MMP expression by the NF-κB and MAPK signaling pathways [138]. In addition, in vivo tests also demonstrated that SPs significantly reduce intracellular ROS levels, cell death, NO production, and lipid peroxidation levels in UVB-irradiated zebrafish in a dose-dependent manner [139]. These results pointed to SPs as potential ingredients in the cosmeceutical industry.

## 12. Other Potential Photoprotective Compounds of Marine Origin

Melanin-related compounds, such as eumelanin from sepia ink, have been proposed as natural UV filters and antioxidants, although their actual use in topical formulations is limited by the unaesthetic dark color conferred to the skin by any product containing eumelanin [140]. Another compound whose photoprotective abilities have recently been investigated is topsentin, a bis(indolyl)imidazole alkaloid, isolated from the marine sponge *Spongosorites genitrix* [141]. It has been shown to inhibit the expression of cyclooxygenase-2, an important enzyme involved in prostaglandin E2 (PGE2) synthesis, and miR-4485, a newly identified inflammation marker in UVB-irradiated human keratinocytes, and lowered PGE2 formation in a reconstructed human skin model [141]. 

Worthy of note is that some cyanobacteria, microalgae, and several marine invertebrates produce other amino acid derivatives such as sulfur-containing histidine endowed with antioxidant properties, and anti-inflammatory and antiproliferative activities in human cells [28,142,143,144,145,146]. They are named ovothiols due to their high concentration in sea urchin eggs [147] and exhibit a characteristic absorbance spectrum (200–320 nm) with a maximum around 257 nm [148]. In addition, the gene expression of the key enzyme involved in their biosynthesis is modulated by several environmental factors, including exposure to metals and toxins in sea urchin embryos [149] and to high light in microalgae [29]. The light-induced modulation of ovothiol biosynthesis suggests their possible role in the protection of microalgae from light-induced stress. This, together with ovothiols’ UV absorption properties, might point to them as potential candidates for photoprotective activities.

## 13. Concluding Remarks and Future Perspectives

The effective dissemination of scientific research through social media has led to increased awareness in the population about the risk of using certain chemicals in drugs and cosmetics as well as the health benefits associated with compounds obtained from natural resources. This has contributed to the expansion of the “blue biotechnology” from the marine environment as opposed to the “green technology” from the terrestrial one. Oceans occupy approximately 71% of the Earth's surface and they represent the “special” environment where the first living forms originated and evolved. Nevertheless, due to their vast extension, human knowledge concerning the sea is limited to a small part of marine environments and organisms, which still provide an extremely rich hotspot of biodiversity in terms of species and bioactive compounds, including natural sunscreens. A few products of marine origin with photoprotective properties have already appeared on the market, as mentioned in this review, but the number of these products is still very minimal considering the vastness of the sea; hence, there is great potential for breakthroughs in the not-too-distant future.

To exploit the potential of the marine environment, more efforts in research and development investments should be dedicated to the isolation and characterization of products from this natural resource, to discover the UV chromophores, and to evaluate their photoprotective properties and skin safety aspects. After all, it is reasonable to benefit from and mimic the sun-screening mechanisms that nature has optimized and refined over thousands of years to withstand sun overexposure. Marine-derived UV filters possess molar absorption coefficient (ε) values typically around or greater than 20,000, compared to the values of synthetic commercial UV filters that usually range from 10,000–30,000 [52], making nature-derived sunscreens particularly appealing and comparable with the man-made counterparts, currently available on the market. Furthermore, some natural compounds also possess broad UVA/UVB spectrum coverage [150] which would reduce the need for blending a series of filters to achieve this purpose. An additional feature in favor of marine-derived photoprotectants is that they are generally regarded as safe, since they are naturally found in seaweed and marine animals without any toxicity, and they have been part of human diets for hundreds of centuries, thus unlikely to be harmful. Moreover, marine-derived sunscreens may represent a possible biodegradable solution to curb environmental pollution coming from the use of all kinds of synthetic cosmetic products, not only sunscreens, which, once disposed and released into the environment, may contaminate ground and sea waters, destabilizing the health of marine habitats and wildlife such as coral reefs. UV filters washed off from sunscreens is another potential source of sea and freshwater pollution since sunscreen formulations are never 100% waterproof. 

Some of the limitations preventing the full exploitation of marine resources for sun-screening purposes are those linked to the low concentrations of these compounds in natural producers, and whose biosynthesis is often influenced by environmental fluctuations, thus offering an overall low yield compared to that obtainable for synthetic products. Another limitation concerns large-scale sourcing or non-sustainable production methods that could disrupt already threatened marine ecosystems. These drawbacks could be overcome by resorting to sustainable, modern aquaculture farming techniques of UV-filter-producing marine sources, coupled with the optimization of growing and harvesting conditions for maximum yields of bioactive compounds for cosmeceutical purposes. Indeed, photosynthetic marine organisms have the inherent ability to self-renew and reproduce, ensuring sustainable supplies. In addition, exciting breakthroughs in this direction are coming from new developments and technical improvements for the cultivation of photosynthetic microorganisms in photobioreactors, such as the high-density long-term cultivation of the green alga *Chlorella vulgaris* [151]. Furthermore, engineering bacteria, yeast or other emerging industrial hosts, such as microalgae, to efficiently produce large quantities of natural sunscreens is another avenue that is being explored to meet market demands [72]. As appealing as this may seem, the downside of engineering microorganisms is that consumers may remain skeptical on using photoprotectants labeled as “natural” that have been obtained through genetic modification of living organisms. 

Regardless of these issues, nature-derived sunscreen agents for sun-care products must first and foremost be both safe and have a beneficial photoprotective effect on human skin; hence, their concentration, compatibility, and stability in a sunscreen formulation must be carefully evaluated. Additionally, they must confer comparable protection from harmful UV rays over the entire exposure time as the currently available organic and inorganic UV filters present on the market. Furthermore, instead of fully replacing current UV filters in a formulation, natural marine-derived photoprotectants could at first be concomitantly added alongside with them, so that the concentrations of current UV filters may be reduced. This solution might lead to optimal synergistic effects since different nature-derived compounds can exert different UV defense mechanisms and combine them with those of synthetic counterparts.

In conclusion, as we move into an era where compounds derived from natural sources are gaining momentum for use in the cosmetic industry for improving skin protection in an effective and eco-conscious way, marine-derived photoprotectants may be a possible solution. In this review, we have outlined the different marine-derived compounds discovered so far, that could potentially be used for this purpose. They would address issues related to their impact on the health of marine habitats whilst providing the essential sun protection that human skin requires to protect it against damage caused by excessive sun exposure. However, further research is greatly needed before marine-derived compounds become effective and biocompatible mainstream ingredients in sun-care products that indeed attract consumers’ interest.
